# Draft Genome Sequence of *Lactobacillus plantarum* Strain WJL, a *Drosophila* Gut Symbiont

**DOI:** 10.1128/genomeA.00937-13

**Published:** 2013-11-21

**Authors:** Eun-Kyoung Kim, Young Min Park, Oun Young Lee, Won-Jae Lee

**Affiliations:** School of Biological Science, Seoul National University and National Creative Research Initiative Center for Symbiosystem, Seoul, South Korea

## Abstract

*Lactobacillus plantarum* strain WJL, a member of the symbiotic gut bacteria, was isolated from the intestine of the fruit fly, *Drosophila melanogaster*. Here, we report the draft genome sequence of *L. plantarum* WJL.

## GENOME ANNOUNCEMENT

*Lactobacillus plantarum* is a Gram-positive, lactic acid-producing bacterium that is found in a variety of environmental niches (1). Interestingly, *L. plantarum* is frequently observed as a member of commensal bacteria in the metazoan gut, ranging from the fly to the human gastrointestinal tract (2, 3). Although this bacterium is generally considered to be a symbiont that induces beneficial effects in its host, the molecular mechanism by which *L. plantarum* impacts host physiology is largely unknown. *Drosophila*, a classical animal model for developmental biology and innate immunity, is now being introduced to the field of gut–microbiota interactions (4). *L. plantarum* strain WJL was originally isolated as a member of the symbiotic bacteria from the *Drosophila* gut (5). This bacterium is capable of colonizing gut epithelia without provoking host gut immunity (6). Recent genetic analyses further showed that *L. plantarum* WJL stimulates the target of sirolimus kinase activity in the host, especially under poor nutritional conditions, to promote systemic larval growth by optimizing integrated hormone signaling pathways (7). In the current study, the genomic information of *L. plantarum* WJL is presented, which is essential to determine the bacterial genes required for a positive impact on the host physiology.

The genome sequence of *L. plantarum* WJL was analyzed using a 100-bp paired-end library (4,567,308 reads) with the Illumina HiSeq 2000 (Illumina, San Diego, CA). The sequence reads were assembled into 102 contigs using the CLC Genomics Workbench 5.1 (CLC bio, Denmark). The total coverage over the whole genome reached ~198-fold. Functional annotation of the predicted genes was performed using the RAST server (8) and COG (9) database. The genome of *L. plantarum* WJL was estimated to be 3,477,495 bp, and 3,410 open reading frames (ORFs) were identified, which include 4 rRNA genes and 71 tRNA genes. Among the 3,410 ORFs, only 2,356 (69%) were identified with predicted functions based on homologies to previously known proteins. The G+C content of the genome is 44.24 mol%. Deducing the sequence information improves the feasibility of performing genetic analyses of this bacterium as well as comparative genomics with other *Drosophila* gut-colonizing lactobacilli, which will provide novel insights into the molecular interactions between lactobacilli and *Drosophila* gut epithelia.

### Nucleotide sequence accession numbers.

The result of this whole-genome shotgun project has been deposited at GenBank under the accession no. AUTE00000000. The version described in this paper is the first version, AUTE01000000.
